# Allocation of forest net primary production varies by forest age and air temperature

**DOI:** 10.1002/ece3.4675

**Published:** 2018-11-14

**Authors:** Xiang Song, Xiaodong Zeng, Dongxiao Tian

**Affiliations:** ^1^ International Center for Climate and Environment Sciences, Institute of Atmospheric Physics Chinese Academy of Sciences Beijing China; ^2^ Collaborative Innovation Center on Forecast and Evaluation of Meteorological Disasters Nanjing University of Information Science & Technology Nanjing China; ^3^ University of Chinese Academy of Sciences Beijing China; ^4^ Beijing Meteorological Information Center Beijing China

**Keywords:** China’s forest, climate, forest age, net primary production partition

## Abstract

Carbon partition among plant parts has a vital influence not only on the growth of individual plants but also on decomposition, carbon and nitrogen sequestration, and plant–atmosphere water exchange. Although many studies have tried to reveal plant growth mechanisms using observational living biomass or the biomass ratio among different organs, knowledge and understanding about carbon partition is still scarce and exists much uncertainty. In this work, a dataset about 1,089 sample plots of natural forests downloaded from the Chinese Ecosystem Research Network (CERN) was used to explore the dependences of net primary production (NPP) partition among foliage, stem and branch, and root on forest age, and mean annual temperature (MAT). The results found that (a) for all forest types, NPP partition had a significant relationship with forest age (*p* < 0.0001), that is, younger plants usually allocated a higher proportion of the NPP to stems, branches, and roots. As plants aged, an increasing proportion of the NPP was allocated to foliage; (b) MAT was negatively correlated with the proportions of the NPP allocated to foliage (*F*_leaf_; %) and roots (*F*_root_; %), while proportions of the NPP allocated to stems and branches (*F*_stbr_; %) were positively dependent on MAT; (c) independent effect analysis demonstrated that forest age had a larger direct influence on *F*_leaf_ and *F*_root_, while MAT was relatively important for *F*_stbr_; and (d) forest age and MAT had a stronger combined effect on NPP allocation for broad‐leaved forests, while for needled‐leaved forests, the influences of forest age and MAT existed large differences among different forest types. This work not only is important for understanding the contribution of climatic factor and forest age on forest NPP partition, but also provides valuable ideas for developing ecological models.

## 1 INTRODUCTION

The process through which plants allocate carbon among different organs is important not only for plant growth (Shvidenko, Schepaschenko, Nilsson et al., 2007b; Shvidenko, Schepaschenko, McCallum et al., 2007a) but also for forest carbon cycling rates and plant–atmosphere water exchange (Aber, Melillo, Nadelhoffer, Pastor, & Boone, 1991). Such processes are usually influenced by forest types (Dube & Mutanga, 2015), structure characteristics (e.g., forest component, age, and density; Li & Liu, 2014; Li et al., 2014; Wang et al., 2014; Zhang, Wang, et al., 2015), climate factors (Wang et al., 2014; Zhang, Song, et al., 2015), and environmental conditions (e.g., soil texture, and altitude) (Wen & He, 2016).

In the current years, many improvements have been achieved in understanding carbon allocation of individual plant, for example, some have described the distribution of biomass in different parts of individual plants (e.g., Poorter et al., 2012; Reich et al., 2014; Dube & Mutanga, 2015; Zhang, Song, et al., 2015), while others have explored the relationships between biomass allocation and forest age (Litton, Raich, & Ryan, 2007; Yuan & Chen, 2010; Zhang, Song, et al., 2015; Zhao et al., 2014), climate or environmental factors (Chen et al., 2015; Luo, Wang, Zhang, Booth, & Lu, 2012; Poorter et al., 2012; Reich et al., 2014; Wang, Fang, & Zhu, 2008; Zhang, Song, et al., 2015). For instance, it is commonly thought that the proportion of trunk biomass significantly increases with age (Xue et al., 2016), the biomass proportions of the branch and foliage decreases (Xue et al., 2016; Zhang, Song, et al., 2015). However, the carbon allocation pattern between above‐ and belowground compartments varies with plantation type and stand age (Chen et al., 2015). King, Giardina, Pregitzer, and Friend (2007) found that the root and shoot ratio of individual trees increased significantly from the sapling to mid‐mature stage, and then dramatically decreased at the old‐forest stage. On the contrary, Li and Liu (2014) demonstrated that carbon allocation to the root system of black locust forest decreased constantly with stand age. When considering the dependence of biomass allocation on climate/environmental factors, it is usually expected that climate is the key factor controlling the spatial distribution of carbon storage (living biomass or biomass fraction) (Liu, Yu, Wang, & Zhang, 2014). As to the sensitivity of carbon allocation to climate, there is still largely unclear knowledge about it. Zhang, Song, et al. (2015) found that for China's forests, climate accounted for 7.7%, 21.4%, 10.0%, and 19.7% of variances in foliage, branch, stem, and root biomass fractions. However, some work found that for forests across the northeast part of China, root biomass was less limited by precipitation than shoot biomass as a result of biomass allocation change (Wang et al., 2008). It might be because the growth of roots is mainly dependent on soil temperature and moisture instead of air temperature and precipitation (Ericsson, Rytter, & Vapaavuori, 1996). Reich et al. (2014) demonstrated that temperature was a better predictor of biomass allocation than moisture availability because the distribution of biomass fraction to roots or foliage was unrelated to aridity, while Chu et al. (2016) used three different approaches to analyze the same dataset, and confirmed that both temperature and precipitation were critical to carbon allocation.

To get closer to the mechanisms of carbon partition, representative datasets distributing in wide range are of crucial importance, but usually difficult to obtain. Fortunately, with increasing field measurements covering various ecosystem types, China has become a key experimental area for terrestrial ecosystems due to its various climate regimes and diverse ecosystems (Fang, Chen, Peng, Zhao, & Ci, 2001; Ni, 2013). China has approximately 175 million ha of forest, which cover approximately 18.21% of the country's land area (Fu et al., 2010), and the forest types range from boreal needle‐leaved and broad‐leaved forests to temperate deciduous broad‐leaved forests and subtropical evergreen broad‐leaved forests to tropical rainforests (Fang et al., 2001). Such terrestrial ecosystems provide a vital carbon sink (Fang et al., 2001; Piao et al., 2009). To date, there has been some work investigating the biogeographical patterns of biomass allocation in China's forests (Luo et al., 2012; Wang et al., 2014; Zhang, Song, et al., 2015).

In this work, a dataset of the proportions of the NPP allocated among different organs from China's forests was used to investigate the relationships between individual allocation strategies and forest age and climate. Different from most of current work, NPP partition data rather than biomass proportions were used in this work, which represented NPP partition rules more directly. This point is very useful to evaluate and develop ecological models because most of the current ecological models describe individual growth by NPP partition. Furthermore, this dataset includes relatively large number of observational data, covering various forest types over China, so this work is an important supplement to related research, and provides some vital clues not only for ecology, but also for evaluations and developments of ecological models (e.g., Dynamic Global Vegetation Models (DGVMs)).

## 2 MATERIALS AND METHODS

### 2.1 Study site and species

**Figure 1:**
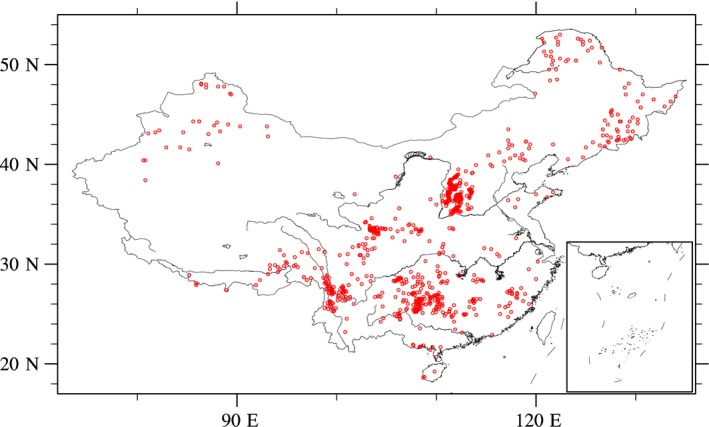
Locations of the 1,089 sample plots of natural forests in the CERN dataset

The observational data used in this work were collected and sorted by Luo (1996), which can be obtained from the Chinese Ecosystem Research Network (CERN) ( https://www.cern.ac.cn/0index/index.asp). It includes 1,089 sample plots of China's natural forests (Figure 1), consisting of longitude, latitude, MAT (°C), mean annual precipitation (MAP; mm/year), annual potential evapotranspiration (PET; mm/year), forest type, age (years), stand population density (stems ha^−1^), biomass (t DM ha^−1^) and NPP (t DM ha^−1^ year^−1^) for each organ (leaf, stem, branch, and root) and stem volume (m^3^ ha^−1^) ( https://159.226.111.42/pingtai/cernc/). This dataset covers 13 forest types, including tropical and monsoon forests, subtropical evergreen broadleaf/coniferous forests, temperate deciduous broadleaf forests, boreal evergreen/deciduous coniferous forests, and so forth (Table 1). The forest stand age ranges from 3 to 350 years, and the MAT and MAP are −6.6 ~ 25.2°C and 25 ~ 3,000 mm/year, respectively.

**Table 1:** Abbreviations for each forest type

Forest type	Abbreviation
Boreal‐temperate deciduous needle‐leaved forest	NDB‐M
Boreal deciduous needle‐leaved forest	NDB
Boreal evergreen needle‐leaved forest	NEB
Temperate evergreen needle‐leaved forest	NEM
Temperate mixed needle‐broad‐leaved forest	*N*‐BM
Temperate deciduous broad‐leaved forest	BDM
Temperate‐subtropical deciduous forest	BDM‐ST
Desert riverside woodland	DerW
Subtropical mixed evergreen‐deciduous broad‐leaved forest	BE‐DST
Subtropical evergreen broad‐leaved forest	BEST
Subtropical montane needle‐leaved forest	MNST
Tropical rainforest and monsoon forest	R‐MT
Subtropical evergreen needle‐leaved forest	NEST

### 2.2 Measurement

The observational NPP involved in this work mainly refers to yearly allocated NPP to stem, branch, foliage, root, as well as bark, and the calculation methods for different organs are usually different. Stem NPP and branch NPP are often estimated based on tree age or growth rate (Luo, 1996). The age method can be described as.

(1) $$\Delta B_{s}=\left(B_{s,a}-B_{s,a-n}\right)/n$$

where ΔB_s_ is the average net growth per area in *n* years, *B*_s, a_ is the existing stem biomass, and *B*_s, a‐n_ is the stem biomass *n* years ago, calculated based on stem analysis. In the other method, the annual woody production of trees is estimated as the woody biomass (stems, branches, and roots) multiplied by the average annual growth rate (%) of the stem volume during the most recent 2 or 5 years (Luo et al., 2004, 2009 ). The species‐specific equations of annual stem growth rate were developed by Luo et al. (2002). The annual production of leaves is estimated as the result of the green leaf mass divided by the species‐specific leaf longevity (Luo et al., 2004, 2009 ). Root net growth rate is usually estimated by the same growth rate as stem (Luo, 1996), that is,

(2) $$\Delta B_{r}=\Delta B_{s}\times B_{r}/B_{s}$$

where Δ*B*_r_ is root net growth per year, *B*_r_ and *B*_s_ are root and stem biomasses, respectively.

### 2.3 Analysis methods

The research objects in this work are the fractions of the annual NPP allocated to individual leaves (*F*_leaf_; %), stems and branches (*F*_stbr_; %), and roots (*F*_root_; %), that is,

(3) $$F_{\text{tissue}}={\text{NPP}}_{\text{tissue}}{\text{/NPP}}_{\text{total}}$$

where NPP_total_ is the total annual NPP per individual and NPP_tissue_ is the annual NPP amount allocated to different organs, that is, leaves, stems and branches, as well as roots. NPP is converted to the forms of carbon content. Sample plots involved in this work are classified into 13 forest types (Table 1), and data are classified into needle‐leaved forests and broad‐leaved forests in some analyses.

Furthermore, to further quantitatively test for direct impacts of climate on NPP, partition independent effect analysis (Chu et al., 2016; Murray & Conner, 2009) is used instead of partial correlations. Following Murray and Conner (2009), independent effect index of variable *x*_1_ (*I_x_*_1_) is defined as.

(4) $$I_{x_{1}}=\sum_{i=0}^{k-1}\frac{\sum \text{(}r_{y,x_{1}x_{h}}^{2}-r_{y,x_{h}}^{2}\text{)}/\left(\begin{array}{c} k-1\\ i \end{array}\right)}{k}$$

where *x*_1_ is the research variable, *x_h_* is any subset of *I* predictors, *x*_1_ excluded.

## 3 RESULTS

### 3.1 Statistics of NPP and NPP partition

**Figure 2:**
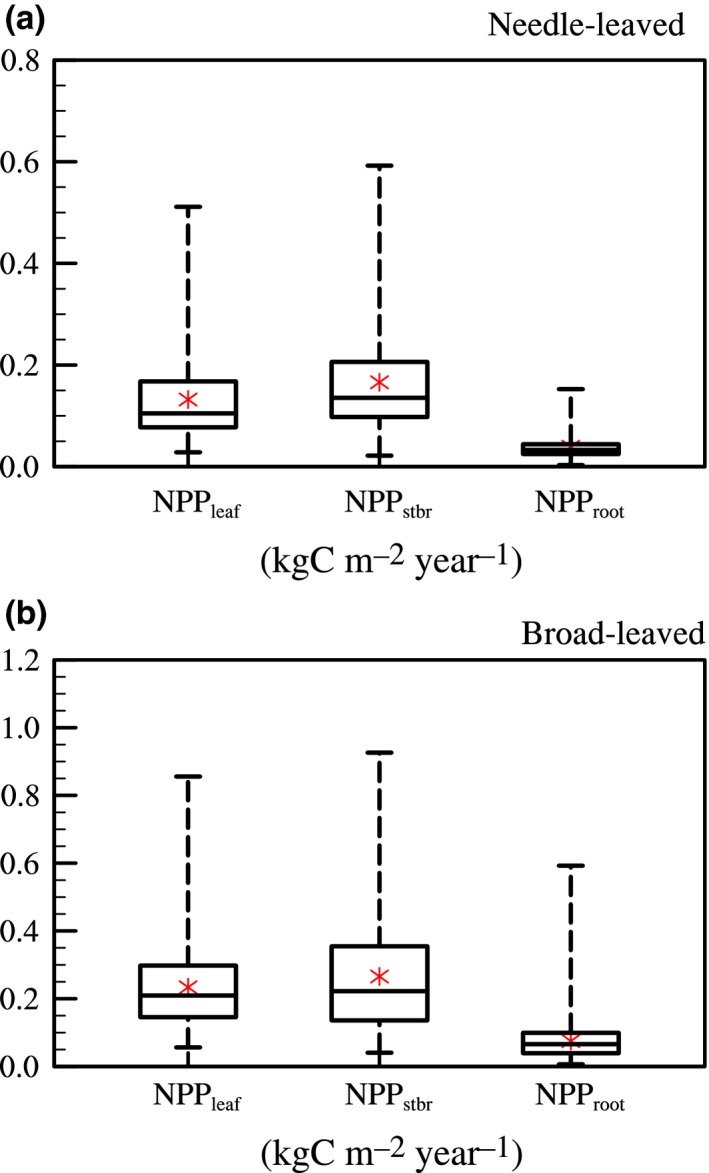
Statistics of net primary production allocated to leaves (NPP_leaf_; kgC m^−2^ year^−1^), stems and branches (NPP_stbr_; kgC m^−2^ year^−1^), and roots (NPP_root_; kgC m^−2^ year^−1^). The black lines refer to the minimum, the 25th%, the median, the 75th%, and the maximum value from bottom to top, and the red star point is the average value for each variable

In this CERN dataset, the averaged NPP for each organ of the broad‐leaved forests was larger than that of the needle‐leaved forests (NPP_leaf_: 0.234 vs. 0.132; NPP_stbr_: 0.266 vs. 0.166; NPP_root_: 0.075 vs. 0.039) (Figure 2). It was probably because broad‐leaved forests usually grew in relatively warm and humid regions, so that photosynthesis effect was stronger than needle‐leaved forests. The variances of NPP allocated to different tissues of the broad‐leaved forests were also obviously larger.

**Figure 3:**
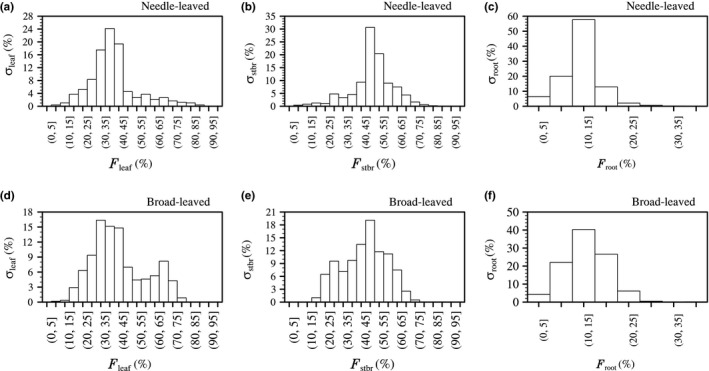
The frequency (*σ*; %) distribution of NPP partition allocated to leaves (*F*_leaf_; %), stems and branches (*F*_stbr_; %), and roots (*F*_root_; %) for needle‐leaved and broad‐leaved forests

Next, we discussed the frequency distributions of the NPP partition proportions (*σ*s; %) (Figure 3). All of the *σ*s were unimodal except the *F*_leaf_ for broad‐leaved forests, and it seemed that the proportions of NPP partition for the needle‐leaved forests were more concentrated in narrow ranges. In the considered plots, the averaged *F*_leaf_, *F*_stbr_, and *F*_root_ were 39.6%, 48.6%, and 11.9%, respectively, for the needle‐leaved forests, while the broad‐leaved forests tended to allocate a higher NPP proportion to foliage (the averaged *F*_leaf_ was up to 47.6%) and a lower NPP proportion to branches and trunks (the averaged *F*_stbr_ was 40.7%). For the needle‐leaved forests, approximately 61.0% of the plots had trees with 30% < *F*_leaf_ ≤ 45%, while for the broad‐leaved forests, plots with 30% < *F*_leaf_ ≤ 45% accounted for approximately 46.3%. Similarly, approximately 61.0% of the needle‐leaved forests and 44.2% of the broad‐leaved forests had trees with 40% < *F*_stbr_ ≤ 55%. The *F*_root_ for the two forest types was mainly below 30%. Approximately 20.0% and 57.7% of the needle‐leaved forest plots had trees with 5% <*F*_root_ ≤ 10% and 10% < *F*_root_ ≤ 15%, respectively, while for broad‐leaved forests, plots with 5% < *F*_root_ ≤ 10%, 10% < *F*_root_ ≤ 15%, and 15% < *F*_root_ ≤ 20% accounted for approximately 22.0%, 40.3%, and 26.6%, respectively.

### 3.2 Factors in relation to NPP partition for China's forests

As is well known, individual growth strategies are governed by a combination of biotic and abiotic factors. Forest age is one of the most important indexes describing plant states, while MAT, MAP and aridity index (the ratio of mean annual precipitation and potential evapotranspiration) (UNEP, 1997) are basic variables depicting temperature and water availability, respectively. However, colinearity diagnostics (eigenvalue analysis and condition index diagnostics) indicated that there was strong colinearity between forest age and MAP (or aridity index), MAT and MAP (or aridity index) in this dataset. Therefore, MAP and aridity index were eliminated from the following statistical analysis.

**Figure 4:**
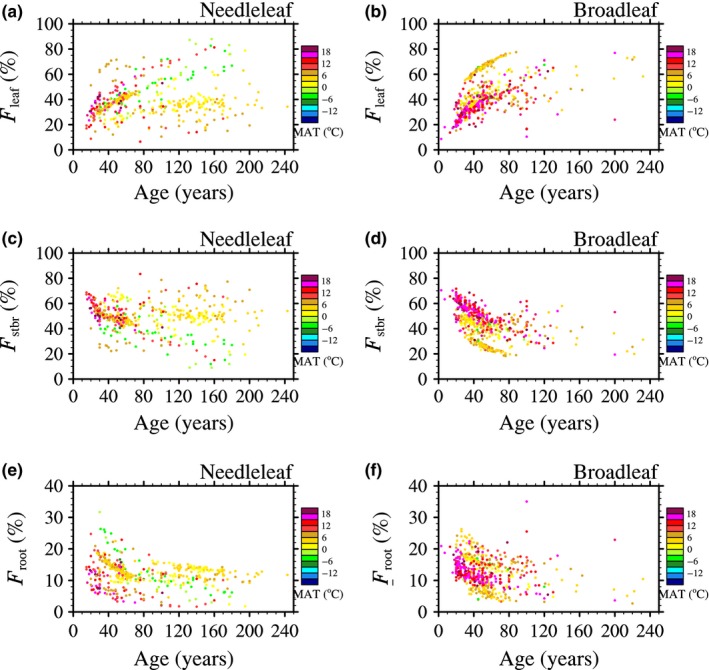
The dependence of NPP partition allocated to foliage (*F*_leaf_; %), stems and branches (*F*_stbr_; %), and roots (*F*_root_; %) on forest age (Age; years) for different temperature zones

First, the relationship between NPP partition and forest age was discussed. In order to exclude the effects of MAT on NPP partition, dataset was divided into several temperature zones (Figure 4). Overall, NPP allocation proportions were significantly sensitive to forest age in all temperature zones (*p* < 0.0001). For both needle‐leaved and broad‐leaved forests, younger individuals were inclined to allocate a lower NPP proportion to foliage and a higher NPP proportion to stems/branches and roots in a given temperature zone (Figure 4). Such phenomena were in accordance with the dynamic behaviors of carbon allocation parameterization in some concept models and numerical models (e.g., aDGVM (Scheiter & Higgins, 2009)): As plants aged, woody biomass becomes sufficient for structure support as well as nutrition and water absorption due to slow turnover rate; therefore, to obtain the maximum photosynthesis rate, an increasing proportion of NPP is used for foliage growth.

**Figure 5:**
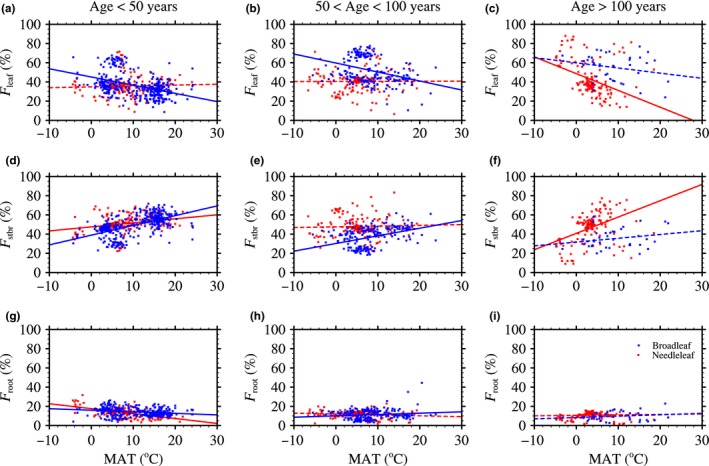
The dependence of NPP partition allocated to leaves (*F*_leaf_; %), stems and branches (*F*_stbr_; %), and roots (*F*_root_; %) on mean annual temperature (MAT; °C) for needle‐leaved and broad‐leaved forests. Solid lines denoted the cases significant at 0.05 level, while the dashed lines mean the opposite cases

Then, to investigate the dependence of NPP partitions on MAT, the dataset was grouped into three age classes: forest age <50 years, 50 ≤ forest age ≤100 years, and forest age >100 years (Figure 5). Results showed that NPP partition had different sensitivities to MAT among various cases. It seemed that NPP allocation proportions of needle‐leaved forests had weak dependence on MAT. However, broad‐leaved forests below 100 years old showed a significant sensitivity on MAT (*p* < 0.0001 for most of cases), and the Pearson correlation coefficient (*R*) between allocation proportions and MAT declined with forest age (Supporting information Appendix S1). Throughout the cases significant at 0.05 level for two forest types, *F*_leaf_ and *F*_root_ decreased with MAT, while *F*_stbr_ increased with MAT (Figure 5).

In nature, the actual plant growth strategies are the emergent properties resulting from interactions among numerous factors. Using independent effect analysis, the results demonstrated that for both needle‐leaved and broad‐leaved forest, forest age played a more significant role in *F*_leaf_ and *F*_root_, while for *F*_stbr_, MAT had dominant impacts. Furthermore, compared with needle‐leaved forests, *F*_leaf_ and *F*_stbr_ for broad‐leaved forests had larger dependences on forest age and MAT (Table 2).

**Table 2:** Independent effect index for NPP partition proportions

	*I*_Age_	*I*_MAT_
Needle‐leaved		
*F*_leaf_	0.027	0.018
*F*_stbr_	0.013	0.065
*F*_root_	0.084	0.056
Broad‐leaved		
*F*_leaf_	0.178	0.147
*F*_stbr_	0.170	0.232
*F*_root_	0.072	0.001

Finally, multiple regressions were used to investigate the combined effects of forest age and MAT on NPP partition (Table 3). It was shown that for both needle‐leaved and broad‐leaved forests, (a) in accordance with Figure 4, *F*_leaf_ had a positive correlation with forest age, while *F*_stbr_ and *F*_root_ had a negative correlation with forest age; (b) *F*_leaf_ and *F*_root_ were negatively correlated with MAT, while *F*_stbr_ was positively correlated with MAT. For needle‐leaved forests, the combined influence of forest age and MAT only accounted for approximately 6.7% and 8.1% of the variances in *F*_leaf_ and *F*_stbr_, while the *F*_leaf_ and *F*_stbr_ of broad‐leaved forests were more remarkably influenced by forest age and MAT which together illustrated 39.8% and 47.1% of the variances in *F*_leaf_ and *F*_stbr,_ respectively.

**Table 3:** Multiple regressions between NPP partition with forest age and climate factors

	Equation	*R* ^2^	*p*
Needle‐leaved			
*F*_leaf_	*F*_leaf_ = 4.344log(Age) – 0.231MAT + 22.401	0.067	<0.0001
*F*_stbr_	*F*_stbr_ = –1.597log(Age) + 0.511MAT + 52.524	0.081	<0.0001
*F*_root_	*F*_root_ = –2.728log(Age) – 0.281MAT + 25.016	0.164	<0.0001
Broad‐leaved			
*F*_leaf_	*F*_leaf_ = 14.693log(Age) – 0.790MAT – 6.036	0.398	<0.0001
*F*_stbr_	*F*_stbr_ = –11.445log(Age) + 0.857MAT + 80.006	0.471	<0.0001
*F*_root_	*F*_root_ = –3.076log(Age) + 24.715	0.100	<0.0001

Note

*F*_leaf_ (%), *F*_srbr_ (%), and *F*_root_ (%) denoted NPP proportion allocated to leaf, stem and branch, as well as root, respectively; Age (years) was forest stand age; MAT (°C) was mean annual temperature.

## 4 CONCLUSIONS AND DISCUSSION

Net primary production allocation among individual foliage, stem and branch, as well as root is thought to be the main mechanism of plant growth. Not only it has a close relationship with forest ecosystem dynamics, but also it has a vital effect on global carbon cycle. As well known, NPP allocation is determined by biotic and abiotic factors together, and there have been a lot of attempts to explore their effects (Chen et al., 2015). However, there is still much uncertainty about NPP partition mechanisms. To identify the effects of forest age and climate, as well as their relative importance for forest NPP partition, independent effect analysis and multiple regressions were used to process the observational data from the Chinese Ecosystem Research Network (CERN) in this work. The results showed that for both needle‐leaved and broad‐leaved forests, (a) NPP partition was remarkably sensitive to forest age, that is, *F*_leaf_ increased with forest age, while *F*_stbr_ and *F*_root_ decreased with forest age. Such the finding is consistent with the observed phenomena that leaf biomass increases in China's subtropical evergreen broad‐leaved forests described in Xiao, Zhou, Zhang, Wang, and Liu (2014) and low root:shoot ratio in current China's forests found by Tang, Zhao, Bai, and co‐authors, (2018); (b) *F*_leaf_ and *F*_root_ were negatively correlated with MAT, while *F*_stbr_ was positively correlated with MAT; (c) independence effect analysis demonstrated that forest age played a more significant role in *F*_leaf_ and *F*_root_, while for *F*_stbr_, MAT had dominant impacts. In addition, compared with needle‐leaved forests, NPP partition of broad‐leaved forests with age <100 years had a stronger dependence on forest age and MAT.

As shown above, the NPP partition of needle‐leaved forests was not very sensitive to forest age and MAT (Table 3). It was probably because of mixture among forest stands with different traits. Previous studies have reported that biomass partition is usually age‐specific (Peichl & Arain, 2007). Similarly, from Figure 5, it was found that MAT had no significant influence on *F*_leaf_ and *F*_stbr_ for needle‐leaved forests with age <100 years, while for stands with age >100 years, *F*_leaf_ decreased with MAT, and *F*_stbr_ increased with MAT, which indicated that the sensitivity of NPP partition to MAT could change with plant growth. On the other hand, there are six forest types grouped into needle‐leaved forests, and NPP partition of different forest types may have diverse sensitivity to forest age and MAT. To test this assumption, multiple regressions between NPP partition and forest age as well as MAT for 13 forest types were calculated, respectively (Table 4). Due to forest type refinement, fitting degree of regression equation was remarkably improved, especially for needle‐leaved forests. For example, the combination of forest age and MAT accounted for about 81.4% and 66.9% of the variance in *F*_leaf_ and *F*_stbr_ for NDB‐M. Meanwhile, for some other needle‐leaved forests, NPP partition was indeed not sensitive to forest age and MAT (*N*‐BM) or was only significantly influenced by forest age (NEB, NEM, and NEST). It also should be noted that for NDB‐M, NDB, and NEB, *F*_stbr_ was significantly and positively correlated with MAT, while *F*_stbr_ of NEST was negatively correlated with MAT. Such distinction of relationship was also likely to reduce the fitting degree of equation in Table 3.

**Table 4:** Multiple regressions between NPP partition with forest age and climate factors for 13 PFTs

PFTs	Equation	*R* ^2^	*p*
*NDB‐M*	*F*_leaf_ = 29.828log(Age) − 1.758MAT − 81.618	0.814	<0.0001
	*F*_stbr_ = −20.845log(Age) + 2.388MAT + 130.907	0.669	<0.0001
	*F*_root_ = −8.985log(Age) – 0.627MAT + 50.752	0.659	<0.01
*NDB*	*F*_leaf_ = 9.857log(Age) − 1.872MAT − 6.403	0.363	<0.0001
	*F*_stbr_ = −8.667log(Age) + 1.682MAT + 89.251	0.295	<0.0001
	*F*_root_ = −1.196log(Age) + 0.184MAT + 17.231	0.068	<0.05
*NEB*	*F*_leaf_ = 17.385log(Age) − 25.783	0.911	<0.0001
	*F*_stbr_ = −11.370log(Age) + 88.110	0.827	<0.0001
	*F*_root_ = −6.104log(Age) + 38.082	0.880	<0.0001
*NEM*	*F*_leaf_ = 13.790log(Age) − 14.595	0.231	<0.05
	*F*_stbr_ = −9.685log(Age) + 85.296	0.174	<0.0001
	*F*_root_ = −4.072log(Age) + 29.157	0.231	<0.0001
*N‐BM*	–	–	>0.4
	–	–	>0.7
	–	–	>0.3
*BDM*	*F*_leaf_ = 21.620log(Age) + 0.747MAT − 33.080	0.262	<0.1
	*F*_stbr_ = −12.849log(Age) + 83.932	0.195	<0.0001
	*F*_root_ = −7.699log(Age) + 40.548	0.302	<0.0001
*BDM‐ST*	*F*_leaf_ = 16.525log(Age) − 21.519	0.542	<0.0001
	*F*_stbr_ = −11.062log(Age) + 84.613	0.488	<0.0001
	*F*_root_ = −5.458log(Age) + 36.897	0.308	<0.0001
*DERM*	*F*_leaf_ = −14.563log(Age) − 75.657	0.400	<0.1
	–	–	>0.2
	–	–	>0.3
*BE‐DST*	–	–	>0.3
	*F*_stbr_ = 56.193	0.071	<0.0001
	*F*_root_ = 1.210MAT	0.311	<0.01
*BEST*	*F*_leaf_ = 15.652log(Age) + 0.446MAT − 30.978	0.607	<0.002
	*F*_stbr_ = −13.876log(Age) + 0.308MAT + 109.348	0.675	<0.005
	*F*_root_ = −1.776log(Age) − 0.140MAT + 21.657	0.054	<0.1
*MNST*	*F*_leaf_ = 23.577log(Age) − 1.174MAT − 34.059	0.580	<0.05
	*F*_stbr_ = −17.646log(Age) + 1.243MAT + 101.430	0.617	<0.0001
	*F*_root_ = −5.924log(Age) + 31.886	0.334	<0.0001
*R‐MT*	–	–	>0.4
	–	–	>0.3
	*F*_root_ = −2.810log(Age) + 0.987MAT + 0.865	0.747	<0.1
*NEST*	*F*_leaf_ = 8.448log(Age)	0.300	<0.001
	*F*_stbr_ = −8.998log(Age) − 0.589MAT + 93.876	0.422	<0.1
	–	–	>0.6

Note

*F*_leaf_ (%), *F*_srbr_ (%), and *F*_root_ (%) denoted NPP proportion allocated to leaf, stem and branch, as well as root, respectively; Age (years) was forest stand age; MAT (°C) was mean annual temperature.

For broad‐leaved forests, it was found that the correlation of NPP partition with MAT declined across the three age classes: It was highest for stands 0–50 years old, but not sensitive for stands above 100 years old. It also may be because forest characteristics significantly change with age, meanwhile, the responses of NPP partition to MAT critically depend on forest composition and structure (Coomes et al., 2014). On the other hand, in older stands with higher NPP, there is a greater likelihood of equilibrium or stand decline, weakening the relationship of biomass change to NPP (Chu et al., 2016; Michaletz, Cheng, Kerkhoff, & Enquist, 2014). So, if using biomass change in short intervals defined as NPP, the relationship between biomass change and climate probably cannot reflect the correlation of NPP with climate.

In addition, there may be some uncertainties resulting from observational data in this work. To investigate plant carbon allocation strategies, choosing the proportions of the NPP allocated to different organs seems better. However, forest NPP cannot be directly measured, and alternatively, NPP is defined as the total new organic matter produced during a specified interval (Clark et al., 2001). Ideally, NPP is the sum of all materials including the following: (a) the amount of new organic matter that is retained by live plants at the end of the interval, and (b) the amount of organic matter that was both produced and lost by the plants during the same interval. However, as mentioned in subsection 2.2, the observational NPP in this CERN dataset omits the litter fall, which may lead to underestimate actual NPP (Chu et al., 2016; Clark et al., 2001), and then results in bias in the intrinsic climate dependency of NPP partition.

This work not only is important for understanding the contribution of climatic factor and forest age on forest NPP partition, but also provides valuable ideas for developing ecological models because the NPP partition scheme is usually the sole part describing the individual growth strategies of plants, and such processes directly determine model simulation performances. Of course, more observational data are still needed to further verify the carbon allocation rules.

## **AUTHORS CONTRIBUTION**

Collected and analyzed data: Xiang Song; conceived paper writing: Xiang Song and Xiaodong Zeng; checked results and polished writing: Xiang Song, Xiaodong Zeng, and Dongxiao Tian.

## DATA ACCESSIBILITY

The data used in this work can be downloaded on the website  https://159.226.111.42/pingtai/cernc/index.jsp after registration and application.
