# Supplementary material for: Allocation of forest net primary production varies by forest age and air temperature
Source: Ecol Evol. 2018 Nov 14;8(23):12163–72. doi: 10.1002/ece3.4675 (PMC6303727; doi:10.1002/ece3.4675)
Supplement: Supplementary file 1 [file ECE3-8-12163-s001.docx]

**Appendix S1.** The correlation between NPP partition and forest age

|  | Age | R | p | b |
| --- | --- | --- | --- | --- |
| Needleleaf |  |  |  |  |
| F_leaf_ | <50 | 0.049 | 0.463 | 0.092 |
|  | 50~100 | 0.006 | 0.055 | 0.012 |
|  | >100 | 0.394 | <0.0001* | -1.748 |
| F_stbr_ | <50 | 0.257 | 0.0009* | 0.420 |
|  | 50~100 | 0.040 | 0.386 | 0.078 |
|  | >100 | 0.444 | <0.0001* | 1.700 |
| F_root_ | <50 | 0.561 | <0.0001* | -0.513 |
|  | 50~100 | 0.134 | 0.091 | -0.092 |
|  | >100 | 0.053 | 0.488 | 0.047 |
| Broadleaf |  |  |  |  |
| F_leaf_ | <50 | 0.426 | <0.0001* | -0.854 |
|  | 50~100 | 0.356 | <0.0001* | -0.938 |
|  | >100 | 0.231 | 0.169 | -0.537 |
| F_stbr_ | <50 | 0.573 | <0.0001* | 1.015 |
|  | 50~100 | 0.409 | <0.0001* | 0.797 |
|  | >100 | 0.231 | 0.169 | 0.395 |
| F_root_ | <50 | 0.229 | <0.0001* | -0.161 |
|  | 50~100 | 0.141 | 0.043* | 0.140 |
|  | >100 | 0.180 | 0.287 | 0.142 |
